# Adenoid Cystic Carcinoma of the Breast: Clinicopathological Features, Therapy Strategy, and Prognosis

**DOI:** 10.1002/cnr2.70442

**Published:** 2026-01-14

**Authors:** Lan Chen, Shuang Dai, Xi Yan

**Affiliations:** ^1^ Fushun People's Hospital ZiGong Sichuan People's Republic of China; ^2^ Department of Medical Oncology West China Hospital, Sichuan University Chengdu Sichuan People's Republic of China; ^3^ Department of Medical Oncology, Head and Neck Cancer Department West China Hospital, Sichuan University Chengdu Sichuan People's Republic of China

**Keywords:** adenoid cystic carcinoma, breast, prognosis, radiotherapy, surgery

## Abstract

**Background:**

Adenoid cystic carcinoma (ACC) of the breast is a special subtype of breast cancer.

**Aims:**

This study aimed to systematically characterize the clinicopathologic features, treatment patterns, and prognostic indicators of breast ACC, with the overarching goal of enhancing clinical understanding and optimizing management strategies for this rare malignancy.

**Methods and Results:**

We conducted a retrospective investigation of 22 patients with breast ACC treated at The West China Hospital of Sichuan University between March 2009 and January 2021. Clinical manifestations, therapy strategies, pathological characteristics, and follow‐up information were systematically observed and analyzed. This study retrospectively analyzed 22 female patients with breast ACC, with ages ranging from 31 to 75 years. Notably, 54.5% (12/22) of the patients were postmenopausal. A palpable breast mass was the most common initial presenting symptom, observed in 95.5% (21/22) of cases. In terms of molecular subtype, triple‐negative breast cancer (TNBC) was identified in 77.3% (17/22) of patients. Conversely, the remaining 22.7% (5/22) of cases showed positive expression for estrogen receptor (ER), progesterone receptor (PR), or human epidermal growth factor receptor 2 (HER2). All patients received surgical treatment, which included simple mastectomy, breast‐conserving surgery (BCS), or modified radical mastectomy. The surgical procedures were optionally accompanied by axillary lymph node dissection (ALND) or sentinel lymph node biopsy (SLNB). The majority of patients, 90.9% (20/22), were diagnosed at early stages (Stage I or II), while only 2 patients were in Stage III. For postoperative adjuvant therapy, 6 patients underwent radiotherapy, 16 received chemotherapy, 2 received hormonal modulation, and 1 advanced‐stage patient participated in a clinical trial with bevacizumab therapy. During the follow‐up period, 4 patients (19%) developed distant metastasis. The 5‐year disease‐free survival rate was 95.4%, with 21 out of 22 patients remaining free from disease recurrence. Remarkably, all patients survived until the end of the last follow‐up, suggesting a relatively good prognosis for breast ACC in this cohort.

**Conclusion:**

Breast ACC is a rare type of TNBC that reportedly has indolent biologic behavior. Definitive preoperative diagnosis remains challenging using imaging modalities alone, as conclusive identification relies heavily on histopathological and immunohistochemical examinations.

**Abbreviations**
ACC: adenoid cystic carcinoma
ALND: axillary lymph node dissection
BCS: breast‐conserving surgery
CNB: coarse needle biopsy
DFS: disease‐free survival
ER: estrogen receptor
FNAC: fine‐needle aspiration cytology
HER2: human epidermal growth factor receptor 2
OS: overall survival
PR: progesterone receptor
SLNB: sentinel lymph node biopsy
TNBC: triple‐negative breast cancer

## 1 Introduction

Primary breast adenoid cystic carcinoma (ACC) is a unique neoplasm subtype, comprising approximately 0.1% of all breast cancer [1]. The majority of breast ACC is classified as triple‐negative breast cancer (TNBC), defined by the absence of estrogen receptor (ER), progesterone receptor (PR), and human epidermal growth factor receptor 2 (HER2). Notably, breast ACC typically exhibits a more favorable prognosis compared to other nonspecific breast cancer subtypes [2, 3]. Reported 5‐year and 10‐year overall survival was 94% and 63.7%, respectively [4, 5]. Due to its rarity, limited studies have characterized the imaging features of breast ACC. Ultrasonography commonly reveals an irregular hypoechoic or heterogeneous mass with an ill‐defined margin. Mammography frequently demonstrates a lobular or irregular shape with indistinct or spiculated margins. Histologically, the tumor is composed of two main cell components: fusiform myoepithelium cells and glandular cuboid cells. Multiple cytoarchitectural patterns (tubular, cribriform, and solid patterns) have been reported, often occurring in combination [6]. Owing to its low incidence and favorable prognosis, consensus has been established regarding the optimal treatment strategy for breast ACC. Surgery remains the primary therapeutic modality. While the role of postoperative adjuvant treatments (chemotherapy, radiotherapy, and hormone therapy) has consistently been a subject of debate.

We conducted a single‐institution retrospective cohort study involving 22 patients with breast ACC. The primary objective of this study was to comprehensively analyze data on clinical manifestations, pathological features, imaging characteristics, and treatment modalities. By systematically evaluating these aspects, we aimed to identify optimal management strategies for breast ACC, thereby enhancing the quality of patient care and clinical decision‐making for this rare malignancy.

## 2 Materials and Methods

This study included patients diagnosed with breast adenoid cystic carcinoma (ACC) at The West China Hospital of Sichuan University between March 2009 and January 2021, with the inclusion criterion being the availability of complete clinical information.

### 2.1 Immunohistochemical Method

All specimens were initially fixed with 4% neutral formaldehyde solution and subsequently subjected to hematoxylin–eosin (HE) staining. Immunohistochemical staining was carried out using the EnVision two‐step technique. The panel of antibodies employed in this study encompassed Ki‐67, ER, PR, HER2, cytokeratin 7 (CK7), CD117, epithelial membrane antigen (EMA), cytokeratin 5/6 (CK5/6), cytokeratin 14 (CK14), cytokeratin 17 (CK17), p63, smooth muscle actin (SMA), among others.

### 2.2 Clinical Date

The information of patients' clinical features (gender, age, menopausal status, clinical presentation, TNM stage, tumor size, imaging characteristics, systemic, etc.), pathological report (ER‐, PR‐, and HER2 status, Ki67, etc.), treatment (surgical management, radiotherapy, chemotherapy, anti‐angiogenesis, and endocrine therapy) was collected.

### 2.3 Follow‐Up

The follow‐up information was obtained through telephone calls, periodic reexamination, or readmission.

### 2.4 Statistical Analysis

All statistical analyses were performed using SPSS 26.0 software (IBM Corporation, Armonk, NY, USA). Cases and percentages calculated patients' basic information. Measurement data that didn't conform to normal distribution were described by median and range. Overall survival (OS) was defined as the interval between the initial diagnosis of the primary tumor by biopsy and the date of death or last follow‐up. Disease‐free survival (DFS) was calculated from the date of the first operation to the first recurrences, local or distant, being scored as an event, and with censoring of other patients at the time of the last follow‐up or death. The Kaplan–Meier estimates were employed to delineate DFS curves.

## 3 Result

### 3.1 Clinical Features

We identified 31 patients diagnosed with breast ACC from the database of 13 425 patients. Twenty‐two patients with available clinical and follow‐up information were selected to enroll in this study. The characters of all patients were shown in Table 1. All patients were female, 12 of them postmenopausal (including one who received a hysterectomy at 37 years old). The diagnosed age range was between 31 and 75 years old (median: 48.5 years old). Only 1 patient has a family history of breast cancer. 95.5% (21/22) primary symptom was a palpable mass, concomitant symptoms including pain in 6 patients, nipple retraction in 3 patients, and bloody discharge in 1 patient. One patient sought medical assistance with a complaint of breast distending pain. Tumors sites included right breast in 18 patients and left breast in 4 patients. The localization of tumor mass in the quadrant was 36.4% (8/22) in the upper outer quadrant, 27.3% (6/22) in the central area, 22.7% (5/22) in the lower outer quadrant, 9.1% (2/22) in the upper inner quadrant, 4.5% (1/22) in the lower inner quadrant.

**TABLE 1:** Clinical characteristics of patients.

Characteristics	*N* (%)
Female	22 (100)
Median age at diagnosed (range), years	48.5 (31–75)
Median tumor size (range), cm	2.55 (1.5–6)
Menstrual status	
Premenopausal	10 (45.5)
Postmenopausal	12 (54.5)
Laterality	
Right	18 (81.8)
Left	4 (18.2)
Location	
Upper outer quadrant	8
Central	6
Lower outer quadrant	5
Other quadrants	3
Family history	
Yes	1 (4.5)
No	21 (95.5)
Lymph node status	
Negative	20 (90.9)
Positive	2 (9.1)
Clinical stage	
I	12 (54.5)
II	6 (27.3)
III	2 (9.1)
Surgery way	
BCS	4
Simple mastectomy	15 (68.2)
Modified radical mastectomy	3 (13.6)
Systemic adjuvant therapy	
Radiotherapy	6 (27.3)
Chemotherapy	16 (72.7)
Hormone therapy	2 (9.1)
Target therapy	1 (4.5)
Histologic subtype	
Cribriform	17 (77.3)
Tubular	4 (18.2)
Solid	1 (4.5)
ER	
−	3 (13.6)
+	19 (86.4)
PR	
−	3 (13.6)
+	19 (86.4)
HER2	
−	19 (86.4)
+	3 (13.6)
Recurrence	
Yes	4 (18.2)
No	18 (81.8)

### 3.2 Pre‐Operative Examination

All patients received axillary and breast ultrasounds, and 9 patients underwent mammograms. The prominent feature was a hypoechoic and/or heterogeneous lobular mass with spiculated or poorly defined margin on ultrasonography. Two patients were found with small calcification in breast mass. Four patients were characterized as axillary nodes with hypoechoic and distinct margins, among which 1 case was confirmed to the lymphatic spread by postoperative pathology. Three patients received the Color Doppler interrogation. One case showed minimal vascularity, and the other two cases showed no color flow. Mammograms of the tumor manifested as unshaped, obscure boundary and asymmetric density. Calcification was presented in three patients.

Twelve patients examined coarse needle biopsy (CNB), two patients experienced fine‐needle aspiration cytology (FNAC), and five patients received excisional biopsy. Two lumps of FNAC and one lump of CNB were considered as intraductal or infiltrating ductal carcinoma. The remaining patients were highly suspected of breast ACC. All patients were confirmed through postoperative pathological diagnosis.

### 3.3 Pathological Characteristics

**FIGURE 1:**
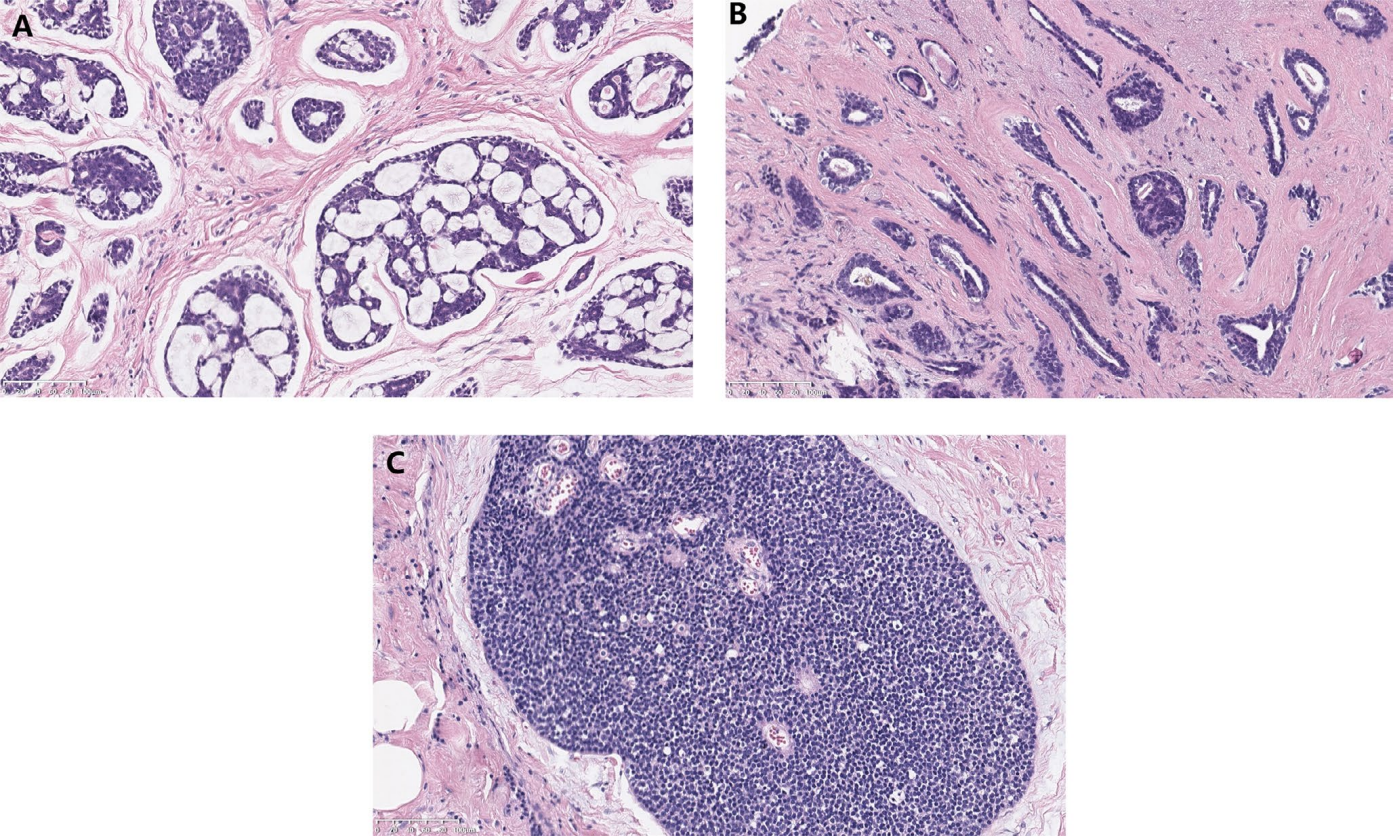
Photomicrograph of tumor specimen shows the typical growth pattern of breast adenoid cystic carcinoma. (A) Cribriform pattern; (B) tubular pattern; (C) basaloid‐solid pattern (H&E staining).

The tumor dimension at the excision ranged from 1.5 to 6.0 cm (mean: 2.55 cm). The gross tumor appeared qualitatively hard, unshaped, not well‐circumscribed, and the cut surface was usually tan‐white or red‐gray with non‐capsule. A total of 90.9% (20/22) of breast ACC were at its early stages (stage I or II), 9.1% (2/22) of patients were at stage III. Microscopically, all cases were pure breast ACC, except 1 that was admixed with the component of squamous cell carcinoma. The tumor nests were mainly composed of spindle myoepithelial and glandular epithelial cells. Dominantly, growth partners of breast ACC included cribriform in 17 cases, tubular in 4 cases, and basaloid‐solid in 1 case (Figure 1A–C).

**FIGURE 2:**
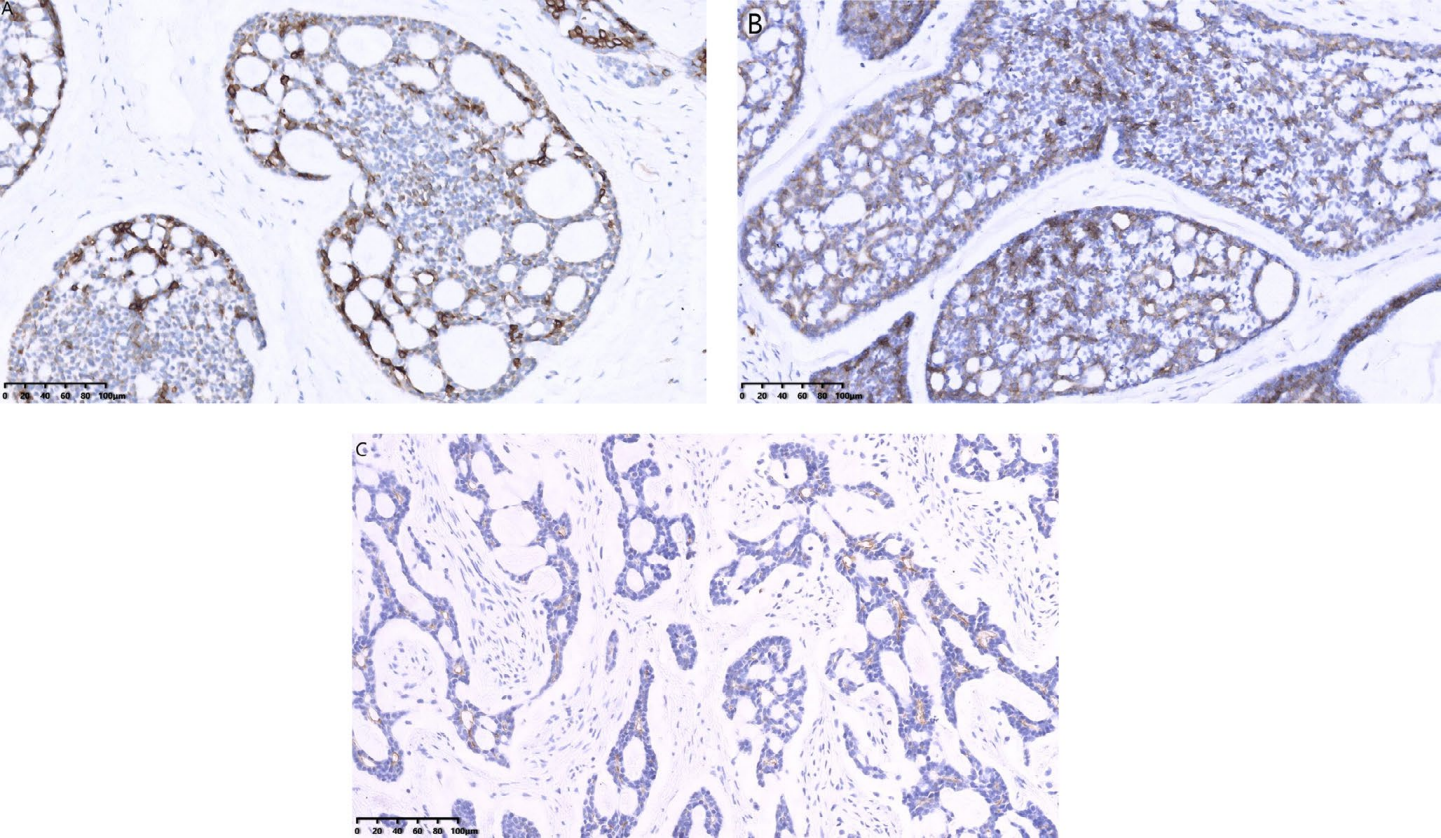
Cytokeratin 7 (A), CD117 (B), and epithelial membrane antigen (C) were diffusely expressed in glandular epithelial cells (EnVision two‐step technique).

**FIGURE 3:**
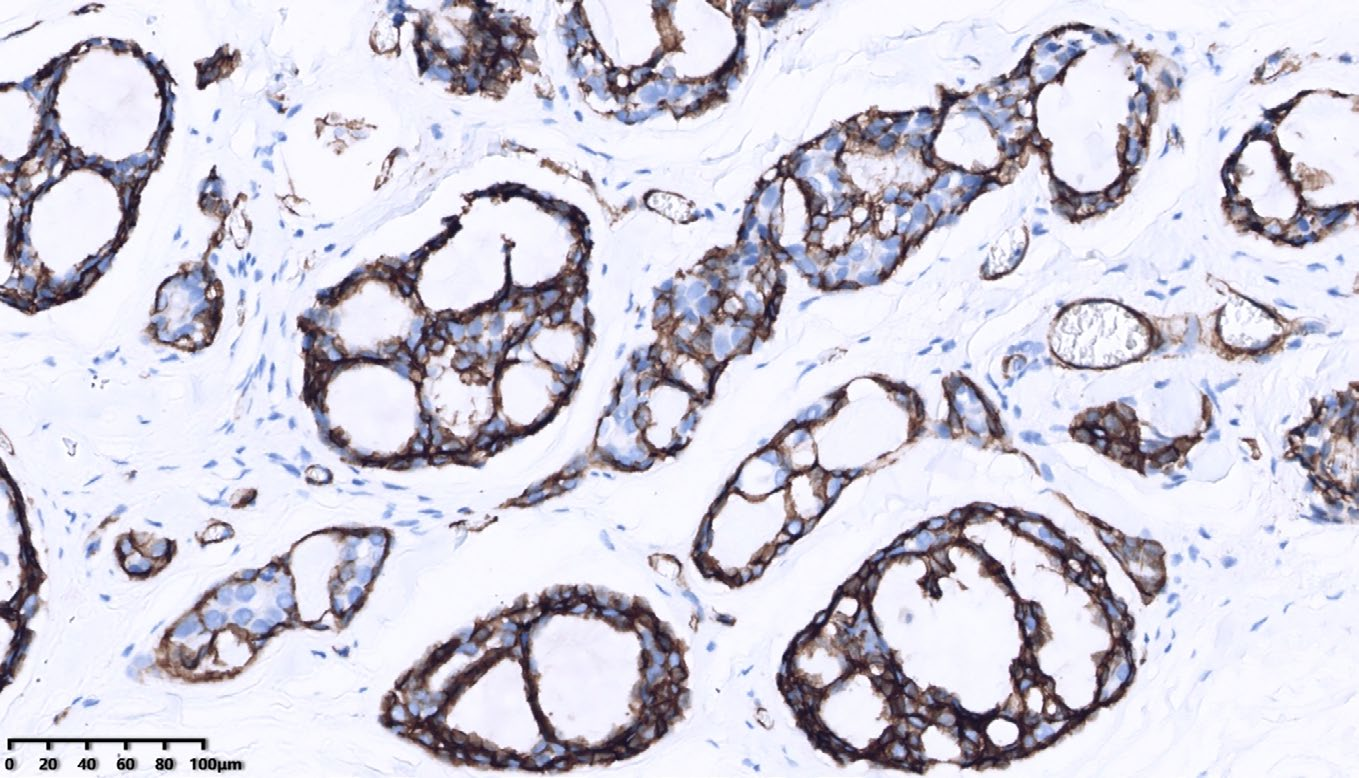
Myoepithelial tumor cells predominantly expressed SMA (EnVision two‐step technique).

**FIGURE 4:**
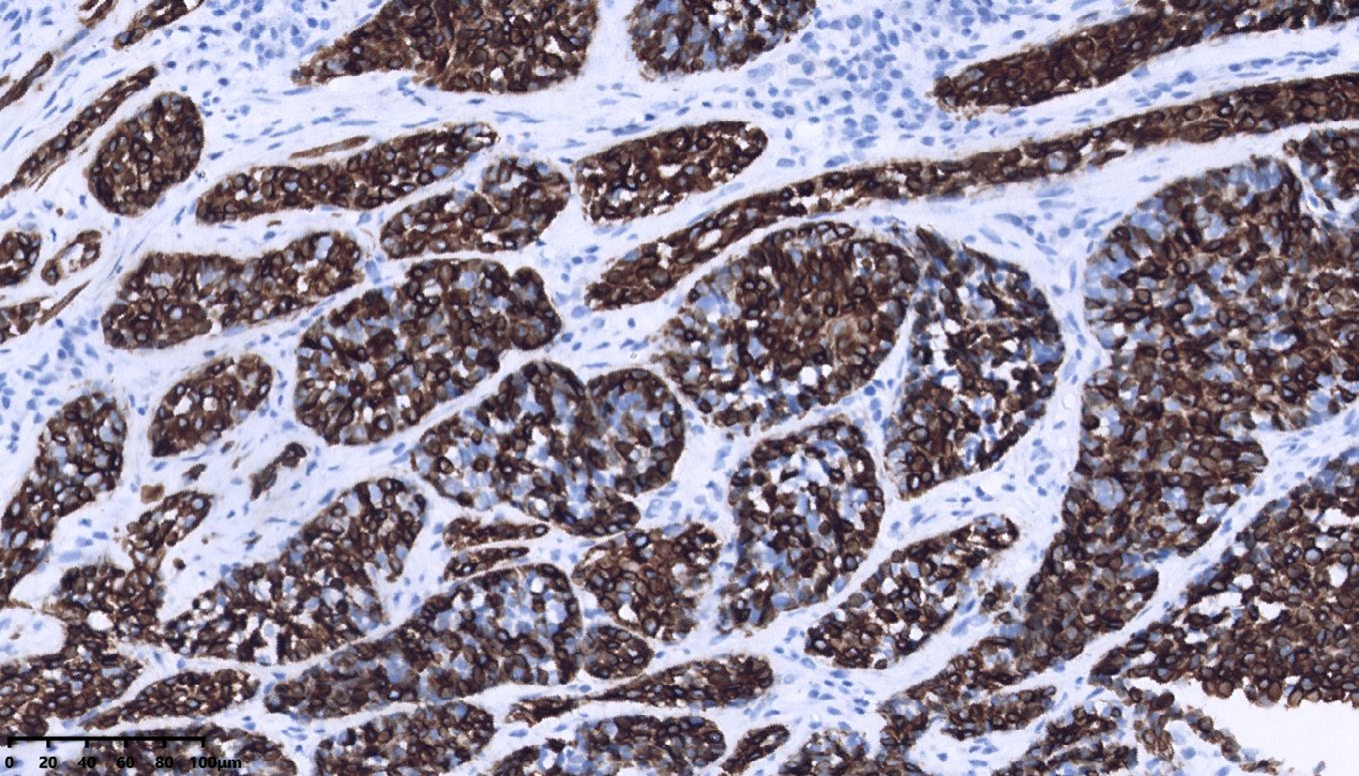
CK5/6 was diffusely expressed in basal cells (EnVision two‐step technique).

One patient had perineural infiltration, and another case had dermal tissue invasion. Two cases of axillary lymph node metastasis, with one and two positive lymph nodes, respectively. The immunohistochemical study was available in all 22 patients. The proportion of patients with ER‐ or PR‐positive status was the same, calculated as 86.4% (19/22). 81.8% (18/22) of patients were HER2‐negative breast cancer. A total of 18.2% (4/22) of patients were HER2(1+). One of them performed with fluorescence in situ hybridization assay was found to have no amplification of the HER2 gene. Patients diagnosed with TNBC account for 77.3% (17/22); 40.9% (9/22) of patients showed a high proliferative fraction with a Ki‐67 labeling index of over 20%, whereas 59.1% (13/22) of patients showed no more than 15%. Furthermore, statistical analysis indicated that in adenoid cystic carcinoma (ACC), glandular epithelial tumor cells frequently demonstrated positive expression of markers such as cytokeratin 7 (CK7), CD117, and epithelial membrane antigen (EMA) (Figure 2A–C). Conversely, myoepithelial tumor cells expressed p63 and smooth muscle actin (SMA) (Figure 3), while basal cells typically exhibited expression of cytokeratin 5/6 (CK5/6) (Figure 4) and p63.

### 3.4 Treatment and Prognosis

All patients received surgery, of which five patients underwent lumpectomy before admission to our hospital. Surgical management of the breast included simple mastectomy in 15 patients, breast‐conserving surgery (BCS) with lumpectomy in 4 patients, and modified radical mastectomy in 3 patients. Eight cases experienced sentinel lymph node biopsy (SLNB), 11 cases underwent axillary lymph node dissection (ALND), 2 cases underwent SLNB + ALND and 1 case received axillary and subclavian lymph node dissection with the exploration of long thoracic and thoracodorsal nerve. 22.7% (5/22) of patients received any postoperative treatment. One patient with ER and PR positive status received prescriptions of Aromasin. The rest of 16 patients received adjuvant chemotherapy, with 5 cases receiving radiotherapy, 1 case receiving hormonal therapy with tamoxifen, and 1 case receiving radiotherapy plus bevacizumab treatment.

**FIGURE 5:**
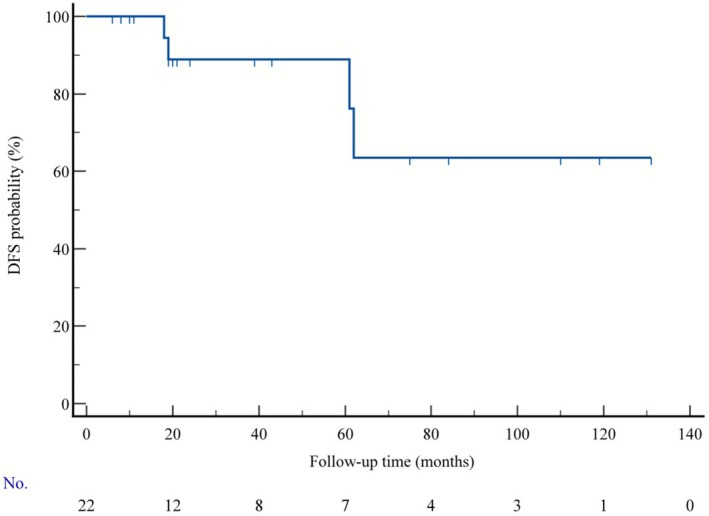
The disease‐free survival (DFS) for 22 patients with ACC of the breast.

The median follow‐up duration of 22 patients ranged from 6 to 134 months (median: 22.5 months). The DFS of all patients was shown in Figure 5. Four patients occurred with pulmonary metastases, one case accompanied with chest and pleural metastasis (Table 2). The time to postoperative recurrence was 61, 66, 134, and 18 months, respectively. The remaining 18 patients were free of disease. Five years of DFS was 95.4% (21/22). All 22 patients were alive at the end of the last follow‐up.

**TABLE 2:** Details of patients with breast ACC.

Patient	Age at diagnosed, (years)	Surgery way	Adjuvant therapy	TNM stage	HER2 status	HR status (ER/PR)	LN status	Ki 67 (%)	Site of metastases (months)	Follow‐up (months)	Status
1	49	M + ALND	C	T1N0M0	1+, FISH (−)	−/−	−	35	−	110	A
2	50	M + ALND	C	T2N0M0	−	−/−	−	20	−	131	A
3	58	M + ALND	C	T2N0M0	−	−/−	−	10	−	119	A
4	50	M + ALND	C	T1N0M0	−	−/−	−	15	−	84	A
5	42	BCS + ALND	C + *R*	T1N0M0	−	−/−	−	10	−	75	A
6	42	M + ALND	C	T1N0M0	−	−/−	−	2	+ lung	61	A
7	52	M + ALND+ SLND+ETTN	C + *R*	T2N1M0	−	−/−	+	20	−	43	A
8	44	M + SLNB	C + H	T1N0M0	+	+/+	−	30	−	24	A
9	35	M + SLNB	No	T1N0M0	+	−/+	−	15	−	8	A
10	43	M + SLNB	C	T1cN0M0	−	−/−	−	15	−	11	A
11	34	BCS + SLNB	C + *R*	T1cN0M0	−	−/−	−	50	−	10	A
12	57	M + SLNB	No	T1N0M0	−	−/−	−	15	−	19	A
13	51	M + ALND+SLNB	No	T2N0M0	−	−/−	−	5	−	20	A
14	43	MRM + ALND+SLNB	C	T1cN0M0	+	−/−	−	15	−	6	A
15	57	M + SLNB	No	T1N0M0	−	−/+	−	5	−	39	A
16	75	M + ALND	No	T4N0M0	−	−/−	−	30	−	21	A
17	48	M + SLNB	H	T1N0M0	−	+/+		3	−	19	A
18	43	MRM + ALND	C + *R*	T1N0M0	−	−/−		10	+ lung	62	A
19	62	MRM + ALND	C	T2N1M0	−	−/−	+	15	+ lung, chest, pleural	18	A
20	51	BCS + ALND	C	T2N0M0	−	−/−	−	15	+ lung	134	A
21	47	M + ALND	C + *R* + T	T2N0M0	−	−/−	−	1	−	21	A
22	31	BCS + SLNB	C + *R*	T1N0M0	−	−/−	−	20	−	20	A

Abbreviations: A, alive; ALND, axillary lymph node dissection; An, anti‐tumor angiogenesis; BCS, breast‐conserving surgery; C, chemotherapy; ETTN, an exploration of long thoracic and thoracodorsal nerve; H, hormonal therapy; M, mastectomy; MRM, modified radical mastectomy; R, radiotherapy; SLNB, sentinel lymph node biopsy.

## 4 Discussion

Adenoid cystic carcinoma (ACC) of the breast is a rare invasive carcinoma that exhibits morphological similarity to its salivary gland counterpart [7]. Compared with salivary gland ACC and other types of breast cancer, breast ACC typically demonstrates a more favorable prognosis, characterized by a lower incidence of tumor recurrence and fewer tumor‐related deaths [8]. However, due to its rarity and the generally favorable prognosis, no consensus has been reached regarding the optimal treatment strategy for breast ACC.

McClenathan and de la Roza identified only 22 patients with breast ACC among almost 28 000 primary breast cancer cases [9]. Most patients diagnosed with breast ACC are postmenopausal, with a median diagnosis age between 50 and 60 years old [10, 11]. Occasionally, young or male patients have also been reported [1, 12, 13, 14, 15]. In our study, we screened out 31 patients diagnosed with breast ACC from nearly 13 500 cases of breast cancer over an 11‐year period. All patients were female, with a median diagnosis age of 48.5 years. Postmenopausal women accounted for 54.5% of the patients (including one patient who underwent hysterectomy at 37 years old). The median diagnosis age in our study was younger than that of foreign patients, which may be associated with the small sample size and ethnic differences. The predominant clinical manifestation prompting hospital visits was a palpable breast mass [4, 16, 17]. Other rare symptoms, including breast pain, nipple discharge, nipple or skin retraction, are less frequent [9, 18, 19]. In our study, one patient initially presented with breast distending pain. The remaining 21 patients sought medical attention due to breast masses, with accompanying symptoms including breast pain or tenderness in 6 patients, nipple retraction in 3 patients, and bloody nipple discharge in one patient. The tumor can affect bilateral breasts, with the most common localization being the outer upper quadrant [19, 20]. In the series of our patients, approximately 1/3 tumor located in the upper outer quadrant, followed by the central area. Other quadrants were less likely to be involved. These results were consistent with the previous study.

The radiological characteristics of breast ACC are generally nonspecific. The tumor typically manifests as a hypoechoic or heterogeneous mass with an ill‐defined border on ultrasonography. Color Doppler interrogation often reveals minimal vascularity [21]. Concerning mammography, breast ACC is often reported as a high‐density lump with a lobular or irregular contour and an obscured margin [21, 22]. Rare radiographic features include asymmetric or mixed density masses and microcalcifications. Additionally, some mammography images resemble benign lesions, showing lobulated, smooth, or unshaped masses [19, 21, 22, 23]. These characteristics align with our findings. Breast magnetic resonance imaging (MRI), a new technique, has been described in isolated case reports. The tumor often appears as an irregular, round, or lobulated mass with an irregular or spiculated margin [18]. On T1‐weighted imaging (T1WI), the tumor typically exhibits low or isointense signals [19, 24]. T2‐weighted imaging (T2WI) manifestations are diverse: large lesions often show distinct heterogeneous high T2WI signals [19, 22, 25], while the tiny lesions present with iso‐intensity T2WI signals [19, 22]. The contrast‐enhanced MRI demonstrated progressive tumor enhancement from the margin to the center over time, which was associated with the tumor stromal components. Due to the limited diagnostic value of radiologic appearances, further exploration of the imaging features of breast adenoid cystic carcinoma (ACC) is warranted in the future.

Histologically, the neoplastic cell nests consist of a biphasic population of myoepithelial and glandular epithelial cells. The growth patterns exhibit morphological diversity, including cribriform, tubular, and solid types. Slodkowska et al. revealed that breast ACC with the feature of > 50% basaloid component had an inferior prognosis and was more likely to have lymph node involvement and distant spread than conventional breast ACC [26]. Some researchers approved that predominately basaloid‐like breast ACC possessed a higher proliferation fraction measured by Ki‐67 [27]. More than half of patients showed a Ki‐67 labeling index of over 20% in our study. The patient with the basaloid component had the highest index. There was no evidence of recurrence or metastasis during follow‐up, which may be associated with the insufficient sample and unsatisfactory follow‐up period. Numerous previous studies have found that breast ACC is generally negative for ER, PR, and HER‐2. But a proportion of research reported positive status in hormone receptors (ER/PR) and/or HER‐2. In accordance with previous literature, 17 in 22 patients in our study were characterized with TNBC. The positive rate of ER, PR, and HER‐2 was 13.6%, 13.6%, and 18.2%, respectively.

Due to its excellent prognosis and low incidence, standardized criteria for the optimal treatment strategy of ACC have not been established. Surgical interventions ranging from local excision to radical mastectomy remain the primary treatment modality [28]. With simple mastectomy being the most commonly reported surgical approach, sentinel lymph node biopsy (SLNB) can help predict the risk of lymph node metastasis and guide decisions regarding axillary lymph node dissection. Breast‐conserving surgery (BCS) is emerging as a viable alternative for select patients with breast ACC. However, Hodgson et al. demonstrated that BCS alone is associated with high positive margin rates [29]. Through multivariate analysis, Slodkowska et al. found that positive surgical margins were significantly predictive of both locoregional recurrence‐free survival (*p* < 0.001) and distant metastasis‐free survival (*p* = 0.002) [26]. Postoperative radiotherapy may enhance tumor control in cases treated with BCS. For example, Khanfir et al., in a study evaluating 61 cases, reported that patients receiving adjuvant radiotherapy after BCS had a significantly better local control rate compared to those treated with BCS alone (95% vs. 83%, *p* = 0.03) [4]. Data from the SEER database analyzed by Gomez‐Seoane et al. further showed that postoperative radiotherapy was associated with improved overall survival in breast ACC patients (*p* = 0.029) [30]. In our study, three patients underwent BCS plus radiotherapy, while one patient received BCS plus chemotherapy; the latter developed lung metastasis 11 years after surgery. Nevertheless, the small sample size and limited long‐term follow‐up in our study preclude definitive conclusions about the role of postoperative radiotherapy.

Axillary lymph node dissection is not routinely recommended for the low incidence of regional lymph node metastasis [4, 9, 31]. In our study, axillary surgery encompassed ALND in 14 cases, SLNB in 10 cases, and subclavian lymph node dissection (SLND) in 1 case. Finally, only two patients were confirmed to be positive for axillary lymph nodes with involvement of 1 and 2 lymph nodes, respectively. Future exploration of the imaging characteristics and needle biopsy features of breast ACC may benefit custom treatment decisions for patients.

The clinical value of adjuvant chemotherapy remains debatable. Some scholars recommended it in patients with high histological grade, axillary lymph node, distant metastasis, or tumor > 3 cm [32]. In our study, 16 patients received chemotherapy; 7 of them received other adjuvant therapy (radiotherapy, hormonal and anti‐angiogenesis treatment). Three of five patients with the tumor dimension > 3 cm and two patients with positive lymph nodes experienced chemotherapy. However, comparison analysis of DFS and OS between non‐ and chemotherapy groups could not be achieved for the lack of randomized control standard. Breast ACC patients seldom adopt hormone therapy for the high possibility of hormone receptor‐negative cells. In our study, two of four patients with ER and/or PR‐positive status received hormone therapy (Aromasin or tamoxifen).

Notably, despite the generally good prognosis, a subset of patients developed recurrence and/or metastasis during the follow‐up period. Metastasis most frequently involved the lungs [4, 33, 34], with reported cases also involving bone, liver, brain, kidney, and skin [5, 34, 35, 36, 37]. Sun et al. demonstrated that patient survival was significantly associated with lumpectomy plus radiation, nodal stage, and histological grade [28]. Slodkowska et al. showed that breast ACC with > 50% basaloid components had a higher propensity for axillary metastases and distant spread compared to conventional pure breast ACC [26]. In this study, four patients developed metastatic spread to the lungs, chest wall, or pleura; three received chemotherapy, and one underwent chemotherapy plus radiotherapy. Patients with T1N0M0 had a disease‐free survival (DFS) exceeding 5 years, while those with T2N1M0 developed multiple metastases within 18 months post‐surgery. All patients remained alive at the final follow‐up. These findings highlight the importance of long‐term follow‐up for patients with breast ACC.

In summary, our study characterized the clinicopathologic features of breast ACC. Most cases were typically triple‐negative breast cancers with an excellent prognosis. BCSplus radiation or simple mastectomy may be suitable for nearly all tumors. The necessity of axillary lymph node dissection requires further evaluation. Larger‐scale studies are needed to clarify the roles of systemic chemotherapy and hormonal therapy.

## Author Contributions

Conception and design, as well as manuscript review: X.Y. Development of methodology: L.C. and S.D. Collection of data and manuscript writing: L.C. and S.D. Data analysis and interpretation: L.C. and S.D. Final approval of manuscript: all authors.

## Funding

The authors have nothing to report.

## Ethics Statement

The studies involving human participants were reviewed and approved by the Ethics Review Board of the West China Hospital, Sichuan University, Chengdu, Sichuan Province, China. Written informed consent for participation was not required for this study in accordance with the national legislation and the institutional requirements.

## Consent

The authors have nothing to report.

## Conflicts of Interest

The authors declare no conflicts of interest.

## Data Availability

Research data are not shared.
